# A simple maximum intensity projection method for measuring proximal femoral anteversion: An observer reliability study

**DOI:** 10.1002/jeo2.70957

**Published:** 2026-10-19

**Authors:** Marco Bechis, Riccardo Faletti, Francesco Pio Papa, Federica Rosso, Davide Blonna, Domenico Lewis Battaglia, Roberto Rossi, Davide Edoardo Bonasia

**Affiliations:** ^1^ Department of Orthopedics and Traumatology Ospedale Maria Vittoria Torino Italy; ^2^ Department of Radiology Candiolo Cancer Institute Torino Candiolo Italy; ^3^ Department of Radiology Ospedale Infantile Regina Margherita Torino Italy; ^4^ AO Ordine Mauriziano, Department of Orthopedics and Traumatology University of Torino Torino Italy

**Keywords:** CT scan, femoral anteversion, femoral torsion, maximum intensity projection (MIP), proximal femoral version measurement

## Abstract

**Purpose:**

Quantifying femoral neck anteversion (FNA) is essential in lower limb torsional deformities. Because of proximal femur anatomy and computed tomography (CT) acquisition, the femoral head and neck are often visualized on different slices, making identification of the femoral‐head–neck axis (FHNA) challenging. This study introduces a new method based on the maximum intensity projection (MIP) function available in most DICOM (Digital Imaging and Communications in Medicine) viewers. By increasing the thickness of the bone segment displayed on a single two‐dimensional image, MIP allows simultaneous visualization of the femoral head and neck, simplifying FHNA identification. To evaluate intra‐ and interobserver reliability of the MIP method and compare it with established techniques. The hypothesis was that MIP would provide superior repeatability and reproducibility than other techniques.

**Methods:**

Between 2019 and 2023, 135 consecutive bilateral lower limb CT angiographies were retrospectively screened. A calculated sample size of 50 patients meeting the inclusion criteria was chronologically enroled. Radiographic measurements were performed at Time 0 and after 30 days by two independent observers following a calibrated training phase. FNA was measured using five methods: Murphy, Hernandez, Waidelich, Yoshioka and MIP. Intraobserver and interobserver reliability were evaluated using a two‐way random‐effects, single‐measures, absolute‐agreement intraclass correlation coefficient [ICC (2,1)] model, alongside standard error of measurement (SEM). For pairwise comparisons of estimated marginal means derived from the linear mixed‐effects model, Tukey's honestly significant difference adjustment was applied. For pairwise comparisons of bootstrap‐derived ICC differences, *p* values were adjusted using the Holm step‐down procedure.

**Results:**

Fifty CT angiographies were analysed. Mean FNA values were significantly different among most methods (Murphy: 18.4° ± 7.2; Waidelich: 24.6° ± 7.4; Yoshioka: 11.5° ± 7.8; Hernandez: 10.7° ± 7.1; MIP: 13.7° ± 6.2) The proposed MIP method demonstrated high intraobserver repeatability (Observer 1: ICC = 0.97; Observer 2: ICC = 0.95) and interobserver reproducibility (ICC = 0.84 for single‐measure absolute agreement). Bland–Altman analysis revealed method‐dependent non‐interchangeability, with systematic differences between techniques.

**Conclusions:**

The MIP technique simplifies identification of the FHNA and demonstrated the highest overall reproducibility among the evaluated CT‐based methods. It could therefore represent a practical approach for improving the standardization of FNA assessment.

**Level of Evidence:**

Level III, diagnostic study.

**Abbreviations**
CT: computed tomography
FHNA: femoral‐head–neck axis
FNA: femoral neck anteversion
MIP: maximum intensity projection
PCA: posterior condylar axis

## INTRODUCTION

Femoral neck anteversion (FNA) is defined as the forward rotation of the femoral neck relative to the posterior surface of the femur at the knee joint and represents a crucial anatomical parameter influencing both hip and knee biomechanics [26].

Abnormalities of the FNA can predispose to different musculoskeletal disorders. An increased FNA, the most common abnormality, is often associated with in‐toeing walking and may predispose to femoroacetabular impingement, extra‐articular hip impingement, hip and knee chondral lesions, patellofemoral pain and instability [5, 16, 22, 23].

While clinical evaluation is essential in identifying torsional deformities, second level imaging is required to accurately quantify the deformity. Cross‐sectional imaging modalities, such as computed tomography (CT) [10, 30] or magnetic resonance imaging (MRI) [2, 27], are commonly used for determining proximal femoral anteversion.

Numerous techniques have been described in the literature for measuring FNA. FNA is commonly measured with the angle between the femoral head–neck axis (FHNA) (a line connecting the centre of the femoral head and the centre of the femoral neck) and the posterior condylar axis (PCA) (the line tangent to the most posterior points of the medial and lateral femoral condyles on the axial plane). Determining the PCA on axial slices of MRI and CT scan is straightforward. On the other hand, identifying the FHNA can be more challenging. In fact, due to the anatomy of the proximal femur and the acquisition of tomography imaging (such as CT and MRI), femoral head and neck lie on different slices. Therefore, defining the FHNA often entails evaluation of multiple slices. For this reason, the FNA measurement techniques described in the literature use different methods to identify the FHNA, including transverse or oblique planes, single or superimposed slices as well as distinct anatomical landmarks [7, 8, 9, 17, 18, 24, 25, 33, 34]. Consequently, recent papers [1, 29, 30, 32] underlined that the challenges in identifying reliable and reproducible anatomical landmarks have often led to arbitrary determinations of the FHNA across published studies [10]. To date, a gold standard measurement technique for FNA has not been determined yet and there is no consensus regarding mean values for FNA in healthy subjects or thresholds for abnormal FNA [1]. This lack of standardization creates confusion between surgeons approaching FNA measurements and indicating derotational osteotomies.

The main goals of the present study were to (1) describe a new measurement method for FNA using the maximum intensity projection (MIP) algorithm applied to standard CT scans and (2) compare it with the previously described methods in terms of intra‐ and inter‐observer reliability. The hypothesis of this study was that MIP method was more practical and reproducible, with better intra‐ and inter‐observer reliability compared with the existing methods.

## METHODS

This study was approved by the local ethical committee (University of Turin; protocol CET00057/2026).

Between January 2019 and December 2023, a total of 135 consecutive bilateral lower limb CT angiographies were obtained at our institution, collected with Picture Archiving and Communication System (PACS) (IMPAX EE; Agfa HealthCare GmbH) and retrospectively evaluated.

All CT angiographies of the lower limbs included in the study were performed for non‐orthopaedic conditions. Cases with complete lower limb CT angiography (from the iliac crest to the malleoli) were eligible for inclusion.

The following exclusion criteria were applied: (1) previous surgeries on the femur or tibia of the examined limb, (2) previous fractures, (3) congenital or acquired deformities, (4) hip or knee joint replacement, (5) end stage arthritis of the hip or knee, (6) amputation of the lower leg/thigh or incomplete illustration of bony landmarks, (7) movement artifacts and (8) segmentary scans of the lower limbs.

In the initial cohort of 135 patients, 51 cases were excluded based on the predefined exclusion criteria. Of the remaining 84 eligible patients, the first 50 consecutive cases were enroled in a strict chronological sequence to satisfy the a priori sample size requirement. No statistically significant differences in age, sex distribution or primary non‐orthopaedic clinical indications were observed between the enroled cohort and the remaining eligible but non‐enroled patients. A comprehensive screening flow diagram illustrating this process is detailed in Supporting Information S1: Figure 1.

Radiological evaluation and measurements were performed at Time 0 and Time 1 (after 30 days) by 1 sports medicine fellowship–trained orthopaedic surgeon and 1 radiologist, both familiar with rotational analyses (M.B. and F.P.P.). Prior to formal data collection, both observers underwent a calibrated training session involving five non‐enroled CT scans to standardize landmark definition and software utilization. Observers were completely blinded to each other's results, to patient clinical data and to their own previous measurements. To mitigate recall bias, the presentation order of the cases and measurement techniques was fully randomized at Time 1 using a computer‐generated random sequence.

### Radiological analysis and measurements

Scans were obtained with a GE (General Electric Company) Healthcare Revolution CT. The scanning procedure was performed using helical acquisitions with a slice thickness of 0.625 mm, both before and after the injection of an organo‐iodinated contrast agent. The tube current was automatically modulated, with a tube voltage set at 100 kV, a pitch factor of 0.6 and a rotation time of 0.8 s.

All examinations were performed in the supine position with the lower limbs completely extended, relaxed and no extra straps applied.

All measurements were performed using Horos OsiriX DICOM (Digital Imaging and Communications in Medicine) viewer (v4.0.0 RC5). The least count of the software was 0.1° for angles and 0.1 mm for distances. To simplify the measurements for the two observers, only right limbs were considered.

The FNA was measured using the following landmarks: the angle between the FHNA (FHNA, known as the line connecting the centre of the femoral head and the centre of the femoral neck) and the PCA (posterior condylar axis, known as the line tangent to the most posterior points of the medial and lateral femoral condyles on the transverse plane). The PCA identification was common to all techniques, using the single axial slice with the condyles showing their maximum extension from anterior to posterior [28].

On the other hand, the FHNA was identified with five different methods (four previously published methods and the new technique described in the present study). The four described methods included (Figure 1):

‐ Murphy's method [18] (Figure 1a)
‐ Hernandez's method [8] (Figure 1b)
‐ Yoshioka's method [34] (Figure 1c)
‐ Waidelich's method [33] (Figure 1d)

**Figure 1:**
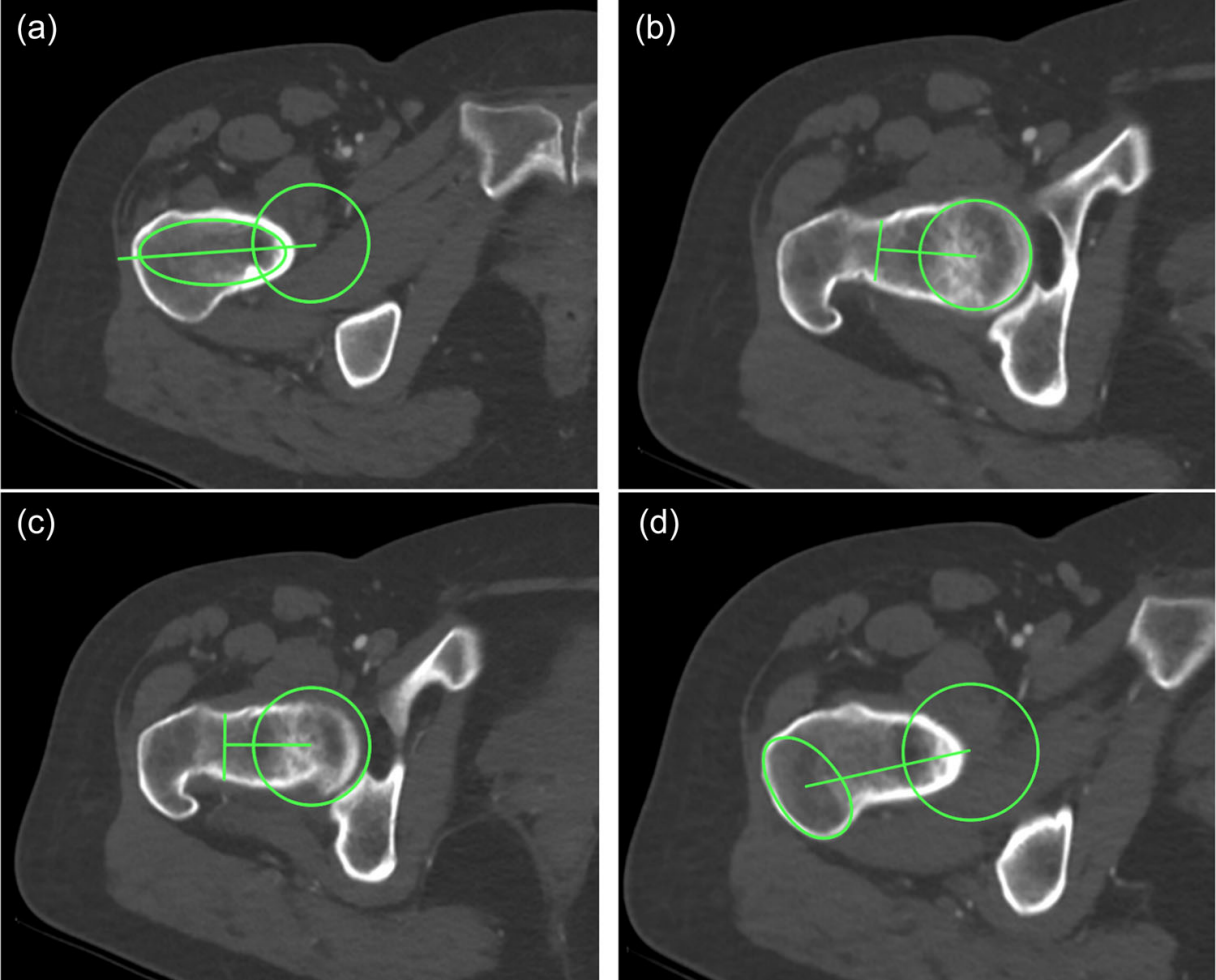
Described methods for femoral‐head–neck axis (FHNA) identification. (a) Murphy's method (superimposed transverse slices): a first slice was used to identify the centre of the femoral head (circle) and a second slice to determine the centre of the base of the femoral neck (centre of an ellipse around the base of the femoral neck). (b) Hernandez's method (single transverse slice): the line between the centre of the femoral head (circle) and the centre of the femoral neck, selecting a single slice in which the femoral head, femoral neck and greater trochanter were all visible. (c) Yoshioka's method (superimposed transverse slices): line running from the centre of the femoral head (first slice) through the midpoint of the narrowest segment of the femoral neck (second slice). (d) Waidelich's method (superimposed transverse slices): line between the centre of the femoral head (first slice) and the centre of an ellipse around the greater trochanter (slice between the tip of the greater trochanter and the lesser trochanter).

Lastly, the FHNA was defined using a new method, using the MIP technique applied to CT scans. The MIP function is common in most DICOM viewers and is an image visualization technique that allows the user to define the thickness and spatial orientation of a volume (slab). Within this slab, for each set of voxels aligned along the specified direction, only the voxel with the highest intensity (or Hounsfield number, representing the greatest density) is selected and depicted so that in a single two‐dimensional image all dense structures in a given volume are observed, independently of their position [20]. In clinical practice this is commonly used to improve the detection of pulmonary nodules and assess their perfusion or to evaluate the size and location of vessels [3, 19].

Given that bone has a high density, the authors considered the use of the MIP visualization technique to achieve a better representation of the complex anatomy of the proximal femur.

After multiple trial measurements, the study group reached a consensus regarding the method that allowed for (1) optimal visualization of the centre of the femoral head together with the femoral neck in a single two‐dimensional (2D) slice and (2) clear identification of the FHNA. The process was standardized through the following sequential steps:

1. *Series selection:* Measurements were strictly performed on the non‐contrast acquisition series utilizing standard bone reconstruction kernels, thereby eliminating any confounding hyper‐intense enhancement from contrast‐filled vascular structures.
2. *Starting slice identification*: The observer scrolled craniocaudally along the unadjusted axial acquisition plane (*Z* axis projection) to identify the most cranial axial slice showing a closed, continuous cortical perimeter of the femoral head.
3. *Slab implementation*: From this baseline slice, a thick‐slab MIP was applied with a standardized slab thickness of 25 mm, projecting strictly perpendicular to the transverse plane without manual tilt or oblique multiplanar reconstruction (MPR) adjustments. Different slabs were evaluated during initial trials: Lower values were frequently insufficient to capture both centres in elongated necks, whereas higher values superimposed the intertrochanteric crest, obstructing neck cortex lines. The 25‐mm threshold was therefore selected to simultaneously capture the centres of both the femoral head and neck within a single 2D projection.
4. *Axis tracing*: On the generated 2D MIP image, the FHNA was defined as the line connecting the geometric centres of two manually positioned circles. First, a circle was manually positioned and adjusted to be tangent to at least three distinct peripheral cortical points of the femoral head. The geometric centre of this circle was then automatically calculated by the software. A second circle was manually positioned at the level of the femoral neck. For femoral necks with a non‐circular configuration, the circle was adjusted to be tangent to the narrowest points of the anterior and posterior cortical margins. The geometric centre of the second circle was subsequently calculated by the software. The line connecting the two calculated centres was defined as the FHNA (Figure 2).

**Figure 2:**
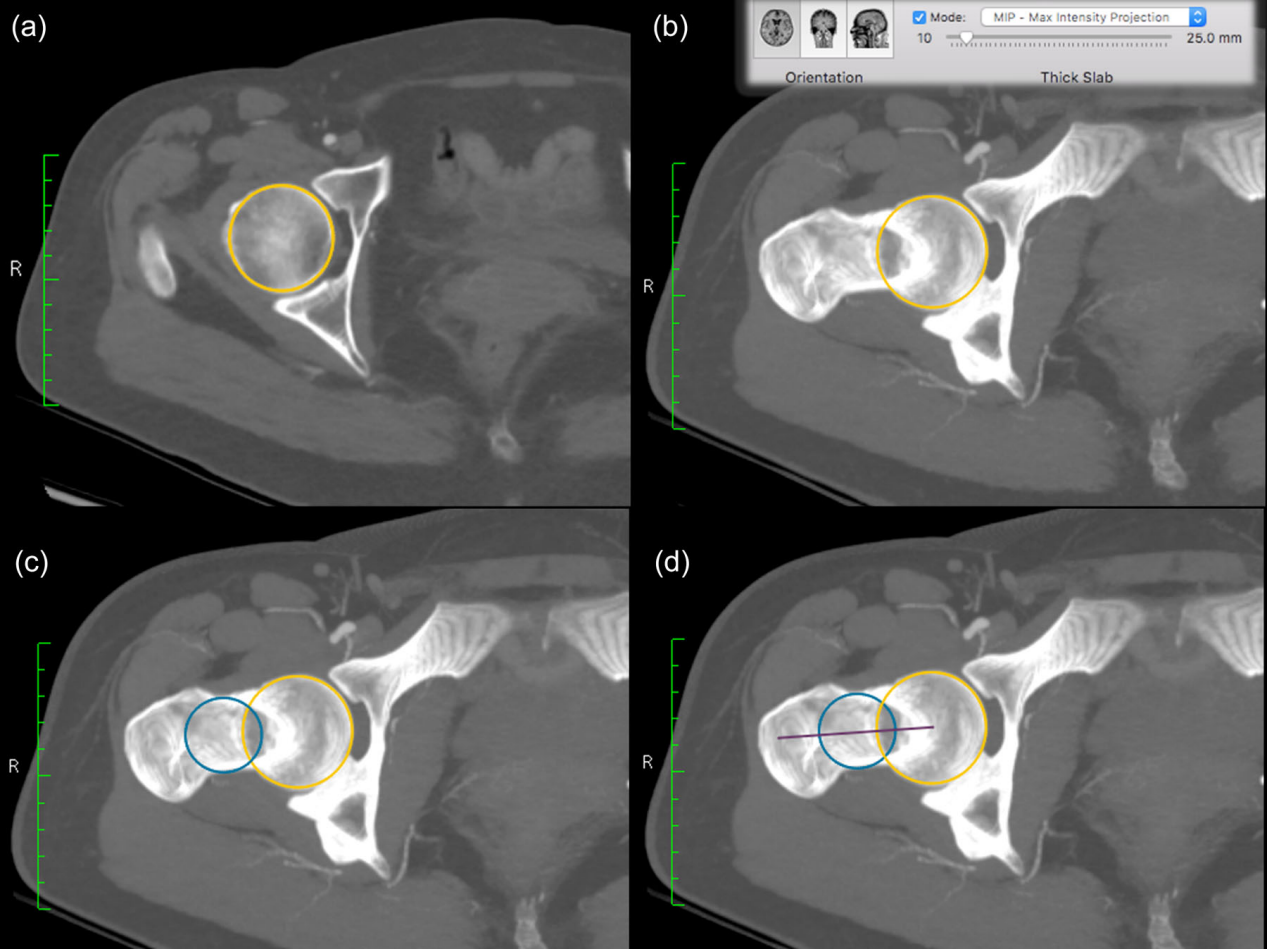
MIP method used for FHNA identification. (a) On the first CT slice with a complete representation of the femoral head the best‐fitting circle of the femoral head was drawn (yellow circle). (b) The MIP function was then implemented. The resulting image showed clear anatomical landmarks required for identifying the FHNA. (c) A second circle touching the anterior and posterior cortices of the femoral neck was drawn (blue circle). (d) The purple line connecting the centres of the two circles was the FHNA. CT, computed tomography; FHNA, femoral‐head–neck axis; MIP, maximum intensity projection.

### Statistical analysis

All radiographic measurements obtained during the two independent assessment sessions were included in the intraobserver reliability analyses. Intraobserver reliability was assessed separately for each observer and measurement technique by comparing measurements obtained during two independent assessment sessions (Time 1 and Time 2).

Reliability was evaluated using the intraclass correlation coefficient [ICC (2,1)] based on a two‐way random‐effects model with absolute agreement for single measurements. A two‐way random‐effects model with absolute agreement was selected in accordance with current recommendations for reliability studies because the aim was to estimate the agreement of single measurements while allowing the reliability estimates to be generalized beyond the specific observers included in this study. Measurement error was quantified using the standard error of measurement (SEM), while agreement between repeated measurements was further evaluated using Bland–Altman analysis by reporting the mean bias and the 95% limits of agreement (LoA).

Interobserver analyses were performed using only the first measurement obtained by each observer (Time 1) to avoid potential carry‐over effects associated with repeated measurements. Interobserver reliability was assessed using ICC (2,1), SEM and Bland–Altman analysis. ICC values were interpreted according to the recommendations of Koo et al. [12], values < 0.50 indicated poor reliability, 0.50–0.75 moderate reliability, 0.75–0.90 good reliability and >0.90 excellent reliability. Ninety‐five percent confidence intervals (95% CIs) were reported for all ICC estimates.

A linear mixed‐effects model was used to investigate the effects of measurement technique and observer on FNA measurements while accounting for clustering of measurements within patients. FNA angle was specified as the dependent variable, whereas measurement technique, observer and the technique × observer interaction were included as fixed effects. Patient was included as a random intercept to account for within‐subject correlation, and model parameters were estimated using restricted maximum likelihood (REML). For pairwise comparisons of estimated marginal means derived from the linear mixed‐effects model, Tukey's honestly significant difference adjustment was applied to control the family‐wise error rate across all pairwise comparisons. For pairwise comparisons of bootstrap‐derived ICC differences, *p* values were adjusted using the Holm step‐down procedure to account for multiple testing. To formally compare the interobserver reliability of the MIP technique with the conventional measurement techniques, patient‐level clustered bootstrap resampling (5000 iterations) was performed. Within each bootstrap sample, interobserver ICC (2,1) values were estimated for all measurement techniques, and the differences between the ICC of the MIP technique and each conventional technique were calculated. Statistical significance was inferred when the bootstrap 95% CI of the ICC difference did not include zero.

A priori sample size calculation was performed for the primary endpoint of interobserver reliability. According to the method proposed by Donner and Eliasziw [6], assuming a minimum acceptable reliability coefficient of 0.60 and an expected reliability coefficient of 0.85, with a two‐sided significance level of 5% and a statistical power of 80% (*α* = 0.05, *β* = 0.20), a minimum of 42 knees was required. Therefore, the inclusion of 50 knees exceeded the minimum required sample size and was considered adequate for the planned reliability analysis.

Statistical analyses were performed using JASP (Version 0.20). Patient‐level clustered bootstrap analyses for comparing ICCs were performed in R (Version 4.5.1).

## RESULTS

Out of 135 consecutive patients, the first 50 CT angiographies meeting the inclusion criteria were enroled in the present study.

The mean age of the patients was 72.4 ± 9.7 years. Thirty‐five patients (70%) were male. The radiographic measurements are summarized in Figure 3 and Table 1.

**Figure 3:**
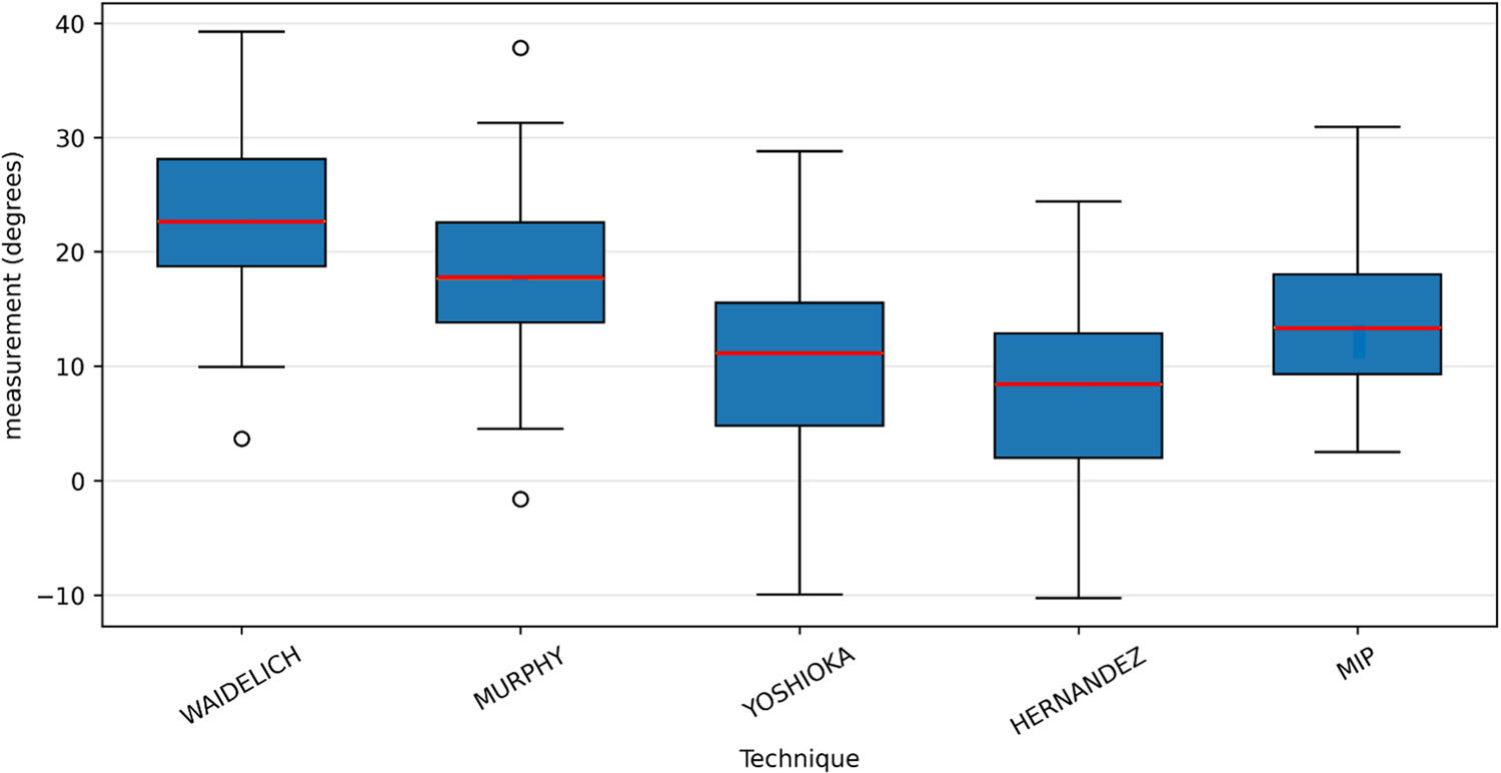
Box‐and‐whisker plot of femoral neck anteversion (degrees) measured with different techniques. The box represents the IQR from the 25th to the 75th percentile; the central horizontal line indicates the median; the whiskers extend to 1.5 times the IQR (or to the minimum and maximum values if no outliers are present); individual points represent outliers. IQR, interquartile range; MIP, maximum intensity projection.

**Table 1:** Femoral neck anteversion values measured with different techniques.

Femoral neck anteversion	Mean (°)	SD (°)
Waidelich	24.6	7.4
Murphy	18.4	7.2
Yoshioka	11.5	7.8
Hernandez	10.7	7.1
MIP	13.7	6.2

*Note*: Values are presented as mean and SD in degrees. Values represent aggregated data averaged across both observers and both sessions (*n* = 200 total measurement entries for each technique).

Abbreviations: MIP, maximum intensity projection; SD, standard deviation.

Mean FNA values differed across the measurement techniques. Waidelich yielded the highest mean anteversion angle (24.6° ± 7.4°), whereas Hernandez produced the lowest values (10.7° ± 7.1°). Murphy (18.4° ± 7.2°) and MIP (13.7° ± 6.2°) showed intermediate values, while Yoshioka (11.5° ± 7.8°) produced measurements similar to those obtained with the Hernandez technique.

### Intraobserver reliability

All measurement techniques demonstrated good‐to‐excellent intraobserver reliability, with consistently higher reproducibility observed for the MIP technique (Table 2). For Reader 1, ICC (2,1) values ranged from 0.748 for the Yoshioka technique to 0.971 for the MIP technique, whereas for Reader 2 they ranged from 0.894 for the Murphy technique to 0.988 for the Yoshioka technique. Corresponding SEM values ranged from 1.08° to 3.45° and from 0.73° to 2.42°, respectively. Bland–Altman analysis confirmed minimal systematic bias for all techniques, with mean differences ranging from −1.13° to 0.33° (Table 2). For Reader 1, the MIP technique demonstrated the narrowest LoA (LoA width, 5.67°), whereas the Yoshioka technique showed the widest interval (19.40°). Similarly, for Reader 2, the MIP technique yielded the narrowest LoA (3.33°), followed by the Yoshioka (4.73°) and Hernandez (5.49°) techniques, whereas Murphy and Waidelich demonstrated wider agreement intervals.

**Table 2:** Intraobserver reliability and agreement of the five femoral neck anteversion measurement techniques for Reader 1 and Reader 2.

Measurement technique Reader 1	ICC (2,1)^a^^,^ ^b^	95% CI	SEM (°)^c^	Bias (°)^d^	95% Limits of agreement (°)^d^	LoA width (°)^d^
Murphy	0.910	0.847–0.948	2.11	0.333	−5.571 to 6.236	11.807
Hernandez	0.815	0.696–0.890	2.76	−0.400	−8.118 to 7.318	15.436
Waidelich	0.872	0.786–0.924	2.51	−0.018	−7.091 to 7.054	14.145
Yoshioka	0.748	0.597–0.849	3.45	0.063	−9.634 to 9.761	19.395
MIP	0.971	0.949–0.983	1.08	−0.194	−3.030 to 2.642	5.672

**Measurement technique Reader 2**	**ICC (2,1)** ^a^ ^,^ ^b^	**95% CI**	**SEM (°)** c	**Bias (°)** d	**95% Limits of agreement (°)**	**LoA width (°)**
Murphy	0.894	0.812–0.940	2.42	−1.129	−7.568 to 5.311	12.879
Hernandez	0.969	0.924–0.985	1.11	−0.765	−3.510 to 1.979	5.489
Waidelich	0.908	0.843–0.947	2.27	−0.822	−7.005 to 5.360	12.365
Yoshioka	0.988	0.976–0.994	0.93	−0.520	−2.883 to 1.842	4.725
MIP	0.987	0.953–0.995	0.73	−0.600	−2.264 to 1.064	3.328

*Note*: Reliability was assessed using the ICC (2,1) and SEM. Agreement was evaluated using Bland–Altman analysis, reporting the mean bias, 95% LoA and LoA width.

Abbreviations: CI, confidence interval; ICC, intraclass correlation coefficient; LoA, limits of agreement; SEM, standard error of measurement.

a Intraobserver reliability was assessed using a two‐way random‐effects, absolute‐agreement, single‐measure ICC(2,1), with 95% CIs.

^b^ ICC interpretation according to Koo and Li (2016): poor (<0.50), moderate (0.50–0.75), good (0.75–0.90) and excellent (>0.90).

^c^ SEM calculated as SD × √(1 − ICC). Lower values indicate smaller measurement error.

^d^ Bias and 95% LoA were derived from Bland–Altman analysis. LoA width was calculated as the difference between the upper and lower LoA and reflects the expected range of variation between repeated measurements.

Overall, although all techniques demonstrated good‐to‐excellent intraobserver reliability, the MIP method consistently showed the highest reproducibility across both readers, combining high ICC values, low measurement error (SEM), minimal systematic bias and the narrowest LoA. Representative Bland–Altman plots illustrating intraobserver agreement for each measurement technique are provided in Supporting Information S1: Figures 2 and 3.

### Interobserver reliability

All measurement techniques demonstrated moderate‐to‐good interobserver reliability (Table 3). ICC(2,1) values ranged from 0.537 for the Hernandez technique to 0.843 for the MIP technique, with SEM values ranging from 2.40° to 4.86°.

**Table 3:** Interobserver reliability and measurement error of the five femoral neck anteversion measurement techniques.

Measurement technique	ICC (2,1)^a^ ^,^ ^b^	95% CI	SEM (°)^c^	Bias (°)^d^	95% Limits of agreement (°)^d^	LoA width (°)^d^
Murphy	0.752	0.602–0.851	3.40	−0.129	−9.682 to 9.425	19.107
Hernandez	0.537	−0.093 to 0.820	4.66	−6.024	−13.840 to 1.790	15.630
Waidelich	0.761	0.447–0.885	4.64	−2.716	−10.620 to 5.183	15.803
Yoshioka	0.577	0.349–0.739	4.86	−2.445	−15.370 to 10.480	25.850
MIP	0.843	0.740–0. 908	2.4	0.441	−6.264 to 7.145	13.409

*Note*: Bias and 95% LoA were obtained from Bland–Altman analysis. LoA width was calculated as the difference between the upper and lower LoA.

Abbreviations: CI, confidence interval; ICC, two‐way random‐effects, absolute‐agreement, single‐measure intraclass correlation coefficient (ICC(2,1)); LoA, limits of agreement; SEM, standard error of measurement.

a Interobserver reliability was assessed using a two‐way random‐effects, absolute‐agreement, single‐measure ICC(2,1), with 95% CIs.

^b^ ICC interpretation according to Koo and Li (2016): poor (<0.50), moderate (0.50–0.75), good (0.75–0.90) and excellent (>0.90).

^c^ Lower values indicate smaller measurement error.

^d^ Bias and 95% LoA were derived from Bland–Altman analysis. LoA width was calculated as the difference between the upper and lower LoA and reflects the expected range of variation between repeated measurements.

Mean bias ranged from −6.02° to 0.44° across the five techniques. The MIP technique demonstrated the narrowest limits of agreement (LoA width, 13.41°), followed by the Hernandez (15.63°) and Waidelich (15.80°) techniques, whereas Murphy (19.11°) and particularly Yoshioka (25.85°) exhibited wider agreement intervals. The MIP (0.44°) and Murphy (−0.13°) techniques showed minimal systematic bias between observers, whereas Hernandez demonstrated the largest systematic difference (−6.02°).

Overall, the MIP technique demonstrated the most consistent interobserver reliability, combining the highest interobserver ICC, the lowest measurement error (SEM), minimal systematic bias and the narrowest LoA.

### Comparison of reliability between measurement techniques

A linear mixed‐effects model demonstrated a significant main effect of measurement technique (*F* = 324.6, *p* < 0.001), indicating that the five techniques yielded significantly different FNA values. A significant observer effect (*F* = 57.4, *p* < 0.001) and a significant Technique × Observer interaction (*F* = 16.1, *p* < 0.001) further indicated that interobserver differences varied according to the measurement technique (Table 4).

**Table 4:** Results of the linear mixed‐effects model evaluating the effects of measurement technique, observer and their interaction on femoral neck anteversion measurements.

Fixed effect	df	*F*	*p* value
Technique	4.432	324.6	<0.001
Observer	1.432	57.4	<0.001
Technique × Observer	4.432	16.1	<0.001

*Note*: Fixed effects were tested using Type III sums of squares with the Satterthwaite approximation for degrees of freedom. Patient was included as a random intercept to account for repeated measurements within subjects.

Abbreviations: df, degrees of freedom; *F*, *F* statistic.

Pairwise comparisons confirmed technique‐dependent observer differences (Table 5). The Hernandez technique showed the largest interobserver discrepancy, with Observer 2 obtaining measurements that were on average 6.02° higher than Observer 1 (*p* < 0.001). Significant observer‐related differences were also observed for the Waidelich and Yoshioka techniques (both *p* < 0.001), whereas Murphy and MIP showed no significant differences between observers, indicating good agreement irrespective of the examiner.

**Table 5:** Pairwise comparisons between observers for each femoral neck anteversion measurement technique estimated from the linear mixed‐effects model.

Technique	Mean difference (1 − 2), °	95% CI	SE	*p* value
Hernandez	−6.02	−7.28 to −4.77	0.64	<0.001
MIP	0.45	−0.81 to 1.71	0.64	0.482
Murphy	−0.12	−1.38 to 1.14	0.64	0.851
Waidelich	−2.71	−3.97 to −1.45	0.64	<0.001
Yoshioka	−2.44	−3.70 to −1.18	0.64	<0.001

*Note*: Estimated mean differences (Observer 1 − Observer 2) were obtained from the interaction between measurement technique and observer. Positive values indicate higher measurements by Observer 1, whereas negative values indicate higher measurements by Observer 2. CIs were calculated at the 95% level using the Satterthwaite approximation for degrees of freedom. *p* values were adjusted according to the model using the Satterthwaite approximation for degrees of freedom.

Abbreviations: CI, confidence interval; MIP, maximum intensity projection; SE, standard error.

These findings are illustrated by the estimated marginal means in Figure 4.

**Figure 4:**
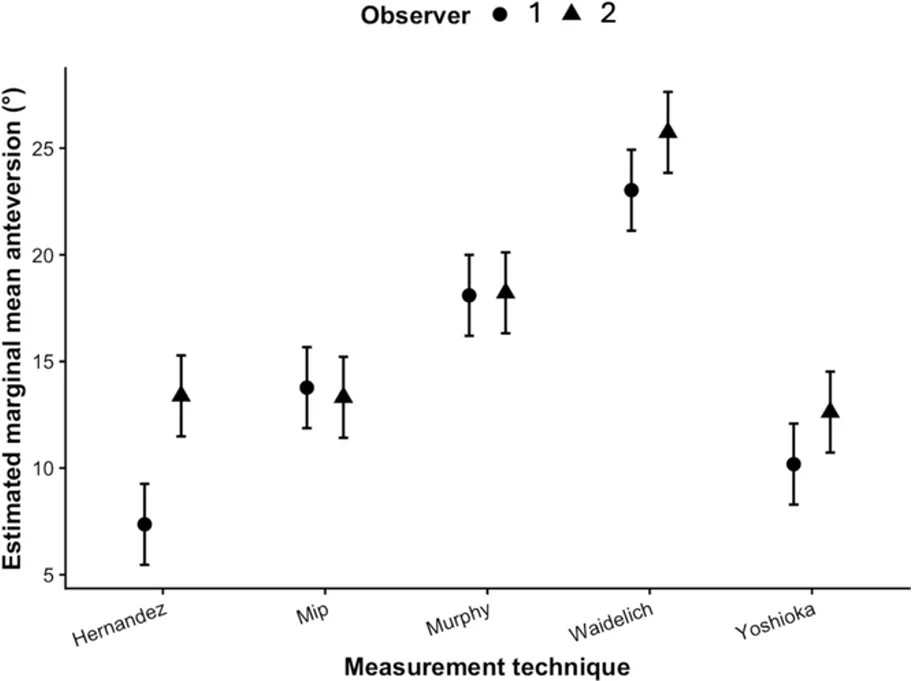
Estimated marginal means of femoral neck anteversion measurements for each technique and observer. Points represent model‐derived estimated marginal means, and error bars indicate 95% confidence intervals. The largest difference between observers was observed for the Hernandez technique, whereas Murphy and MIP showed closely overlapping estimates. Estimates were derived from the linear mixed‐effects model including technique, observer and their interaction as fixed effects and patient as a random intercept. MIP, maximum intensity projection.

To compare overall reproducibility across techniques, a second linear mixed‐effects model using the absolute interobserver difference as the dependent variable demonstrated a significant effect of measurement technique on interobserver error (Type III analysis of variance: *F* = 12.04, df = 4192; *p* < 0.001). Estimated marginal means (Table 6) identified the MIP technique as having the lowest interobserver error, followed by Murphy and Waidelich, whereas Hernandez demonstrated the greatest variability.

**Table 6:** EMMs of the absolute interobserver difference for each femoral neck anteversion measurement technique derived from the linear mixed‐effects model.

Technique	Estimated interobserver error (°)	95% CI
MIP	2.02	0.94–3.10
Murphy	3.55	2.47–4.63
Waidelich	3.58	2.50–4.66
Yoshioka	4.49	3.41–5.57
Hernandez	6.09	5.01–7.17

*Note*: The model included technique as a fixed effect and patient as a random intercept to account for repeated measurements within subjects. Values represent the estimated absolute measurement error (degrees) with corresponding 95% CIs.

Abbreviations: CIs, confidence intervals; EMMs, estimated marginal means; MIP, maximum intensity projection.

Post hoc pairwise comparisons of estimated marginal means were performed using Tukey's honestly significant difference adjustment for multiple comparisons (Table 7) and confirmed significantly greater interobserver error for Hernandez than for MIP, Murphy and Waidelich (all *p* < 0.001). In addition, MIP demonstrated significantly lower measurement error than Yoshioka (*p* < 0.001). No significant differences were observed between MIP and either Murphy or Waidelich after adjustment for multiple comparisons.

**Table 7:** Tukey‐adjusted pairwise comparisons of estimated interobserver absolute error between femoral neck anteversion measurement techniques obtained from the linear mixed‐effects model.

Comparison	Mean difference (°)	95% CI	*p* value
Hernandez versus MIP	4.07	2.40 to 5.75	<0.001
Hernandez versus Murphy	2.54	0.86 to 4.21	<0.001
Hernandez versus Waidelich	2.51	0.84 to 4.19	<0.001
Hernandez versus Yoshioka	1.60	−0.08 to 3.27	0.069
MIP versus Murphy	−1.53	−3.21 to 0.14	0.090
MIP versus Waidelich	−1.56	−3.23 to 0.12	0.081
MIP versus Yoshioka	−2.47	−4.15 to −0.80	<0.001
Murphy versus Waidelich	−0.02	−1.70 to 1.65	1.000
Murphy versus Yoshioka	−0.94	−2.61 to 0.74	0.536
Waidelich versus Yoshioka	−0.91	−2.59 to 0.76	0.562

*Note*: Mean differences are expressed as the difference between estimated marginal means of the first and second technique. CIs and *p* values were calculated using the Satterthwaite approximation for degrees of freedom.

Abbreviations: CIs, confidence intervals; MIP, maximum intensity projection.

Regarding MIP technique, bootstrap analysis confirmed the highest observed reliability among the evaluated methods (Table 8). MIP demonstrated significantly higher ICC values than both Hernandez (ΔICC = 0.306; Holm‐adjusted *p* = 0.004) and Yoshioka (ΔICC = 0.266; Holm‐adjusted *p* = 0.010), whereas no significant differences were observed between MIP and either Murphy or Waidelich after correction for multiple comparisons. Hernandez also demonstrated significantly lower reliability than both Murphy and Waidelich. Overall, bootstrap analysis independently corroborated the findings of the linear mixed‐effects models, consistently identifying the MIP technique as the most reproducible method across observers.

**Table 8:** Bootstrap comparison of interobserver reliability among femoral neck anteversion measurement techniques.

Comparison	ΔICC	95% CI	Holm‐adjusted *p* value
Hernandez versus MIP	−0.306	−0.442 to −0.186	0.004
Hernandez versus Murphy	−0.214	−0.348 to −0.075	0.025
Hernandez versus Waidelich	−0.224	−0.349 to −0.108	0.004
Hernandez versus Yoshioka	−0.040	−0.255 to 0.154	1.000
MIP versus Murphy	0.091	0.004–0.195	0.204
MIP versus Waidelich	0.081	0.001–0.176	0.204
MIP versus Yoshioka	0.266	0.081–0.429	0.010
Murphy versus Waidelich	−0.010	−0.094 to 0.067	1.000
Murphy versus Yoshioka	0.174	−0.012 to 0.318	0.216
Waidelich versus Yoshioka	0.184	0.018–0.325	0.178

*Note*: Pairwise differences in ICC [1,2] were estimated using 5000 bootstrap resamples. CIs were calculated using the percentile bootstrap method. *p* values were adjusted for multiple pairwise comparisons using the Holm procedure. Positive ΔICC values indicate higher interobserver reliability for the first technique listed in each comparison, whereas negative values indicate higher reliability for the second technique.

Abbreviations: CI, confidence interval; ICCs, intraclass correlation coefficients; MIP, maximum intensity projection.

## DISCUSSION

The principal finding of the present study was that the newly proposed MIP technique demonstrated the highest overall reliability for FNA assessment among the five evaluated methods. Although all techniques showed good‐to‐excellent intraobserver repeatability and moderate‐to‐good interobserver reliability, MIP consistently achieved the highest ICCs, the lowest measurement error, minimal systematic bias and the narrowest LoA. Moreover, linear mixed‐effects analyses demonstrated that the choice of measurement technique influenced not only the measured anteversion values but also observer‐dependent variability. Taken together, these findings suggest that simplifying identification of the FHNA improves measurement reproducibility.

FNA is a key anatomical parameter involved in hip and knee biomechanics and plays an important role in the pathogenesis of a wide spectrum of disorders such as gait impairment, chronic hip or knee pain, patellofemoral instability and it can also contribute to the overall hip stability phenotype and influence the interpretation of microinstability and capsular management during hip arthroscopy [5, 16, 21, 22, 23]. Nevertheless, considerable heterogeneity still exists regarding measurement techniques and reference values [1, 26].

The favourable performance of the MIP technique is likely explained by its simplified identification of the FHNA. Unlike conventional CT methods, which require observers to integrate landmarks across multiple axial slices, MIP enables simultaneous visualization of the femoral head and neck on a single reconstructed image. This reduces the need for repeated slice matching while preserving the same anatomical landmarks used by conventional techniques. Rather than introducing a new measurement method, MIP simplifies visualization of the existing anatomy, thereby reducing observer‐dependent variability. Importantly, this simplification can be achieved using MIP reconstructions that are readily available in most modern DICOM viewers, without requiring dedicated software or additional image acquisition.

This interpretation is supported by the physical characteristics of MIP, which reconstructs a volumetric slab by displaying the highest‐density voxels into a single image [20]. Although widely used in vascular and musculoskeletal imaging [3, 19], this is, to our knowledge, the first application of MIP to femoral version assessment. Interestingly, the improvement in reproducibility was observed both within and between observers, suggesting that the technique may facilitate greater measurement standardization across different specialties.

Our findings are consistent with previous studies showing that different CT‐based techniques are generally reproducible but cannot be considered interchangeable. Kaiser et al. [10] demonstrated that six commonly used techniques produced significantly different anteversion values despite acceptable reliability, while Liodakis et al. [15] and Vorimore et al. [31] similarly reported satisfactory reproducibility across different methods. Our results confirm these observations while extending them by demonstrating that measurement technique directly influences observer‐dependent variability and absolute measurement error. Importantly, the highest reliability of the MIP technique was consistently demonstrated across multiple complementary statistical approaches, including ICC, SEM, Bland–Altman analysis, linear mixed‐effects modelling and bootstrap comparison of ICC estimates, strengthening the robustness of the observed findings. Furthermore, our findings support previous evidence that different measurement techniques produce substantially different anteversion values.

Kaiser et al. [10] reported differences of up to 16° between techniques, whereas Vanhove et al. [30] and Lerch et al. [13] emphasized that femoral version should always be interpreted according to the specific measurement method employed. Consistent with these observations, our linear mixed‐effects analysis demonstrated that measurement technique systematically influences the measured anteversion angle. In our cohort, the difference between Waidelich and Hernandez measurements approached 14°, despite both methods showing good reliability, highlighting that methodological variability may substantially contribute to differences reported across the literature. Methods like Hernandez's [8] relied on a single slice at the femoral neck level and could be difficult to use in cases of coxa valga. Conversely, the Waidelich et al. [33] or Murphy et al. [18] techniques utilize multiple slices, making them more complex, demanding and time consuming.

This substantial methodological variability has critical clinical consequences. Currently, the exact same patient's torsional profile could be classified as normal, externally rotated or internally rotated depending solely on the imaging modality, measurement technique and reference thresholds chosen. In this scenario, the thresholds of normality for FNA (10°–25°) described in the literature should be taken with caution [1, 14]. Although the improvement in reliability observed with MIP compared with established methods such as Hernandez was quantitatively modest, even small reductions in measurement variability may become clinically relevant when femoral version values lie close to thresholds used to guide surgical decision‐making. The principal advantage of MIP is therefore not that it changes surgical indications by itself, but that it provides greater confidence that the measured anteversion reflects true anatomy rather than observer‐dependent variability. Together with previous observations by Kaiser et al. [10], Vanhove et al. [30] and Lerch et al. [13], our findings further support the need to report the measurement technique whenever femoral version values are used to establish normative databases, compare clinical studies or define indications for femoral derotational osteotomy.

The present study is not without limitations. First, our cohort consisted primarily of elderly Caucasian individuals and was not designed to establish normative reference values. Femoral version has been reported to vary according to sex and possibly ethnicity [4, 11, 13]. Moreover, the typical clinical candidates requiring accurate FNA quantification for rotational assessment are children, adolescents or young adults presenting with patellofemoral instability or hip impingement. In elderly cohorts, the presence of marginal osteophytes, subchondral sclerosis and osteoarthritic structural remodelling may obscure cortical boundaries, potentially altering the circle‐fitting process. While sharper cortical lines in younger patients might theoretically improve landmark definition, open or transforming physes could introduce confounding factors. Consequently, the reliability metrics and absolute values reported in this study should not be considered as reference values for the general population, since this was outside the scope of the present study, and future diagnostic validation studies within other populations are warranted. Second, although MIP was compared with four of the most widely adopted CT‐based techniques, not every published method was evaluated. Moreover, this study evaluated measurement reliability rather than anatomical accuracy. As no universally accepted gold standard for femoral version exists, future cadaveric and three‐dimensional validation studies are required to determine whether the improved reproducibility of MIP also translates into greater measurement accuracy. Third, although the MIP function is common to most DICOM viewers, in older software it might not be available, limiting the general applicability of the new technique. Fourth, because both observers were highly specialized (a sports fellowship‐trained orthopaedic surgeon and a dedicated radiologist), we acknowledge that high reliability may reflect expert performance. Reproducibility may express lower values among residents or non‐specialized general practitioners. Finally, only CT imaging was assessed, and whether the same advantages apply to MRI remains unknown [2]. Applying a similar algorithmic projection to isotropic 3D T1‐ or T2‐weighted MRI sequences could potentially transfer the reproducibility advantages observed in this CT pilot study to a radiation‐free imaging platform.

## CONCLUSIONS

The MIP technique demonstrated the highest observed reliability among the evaluated methods for measuring FNA. Although the absolute differences in reproducibility were modest, the reduction in observer‐dependent variability may improve confidence in femoral version assessment, particularly when measurements are used to guide clinical decision‐making. Given its simplicity, reproducibility and broad availability, the MIP technique may represent a useful approach toward greater standardization of femoral version measurement across clinical practice and research.

## AUTHOR CONTRIBUTIONS

All authors contributed to the study conception and design. Material preparation, data collection and analysis were performed by Marco Bechis, Francesco Pio Papa and Domenico Lewis Battaglia. The first draft of the manuscript was written by Marco Bechis and Federica Rosso, and all authors commented on previous versions of the manuscript. All authors read and approved the final manuscript. *Conceptualization*: Davide Edoardo Bonasia and Riccardo Faletti. *Methodology*: Davide Edoardo Bonasia and Federica Rosso. *Formal analysis and investigation*: Marco Bechis, Francesco Pio Papa and Domenico Lewis Battaglia. *Writing—original draft preparation*: Marco Bechis. *Writing—review and editing*: Marco Bechis and Davide Edoardo Bonasia. *Supervision*: Davide Edoardo Bonasia, Federica Rosso, Riccardo Faletti, Davide Blonna and Roberto Rossi.

## CONFLICT OF INTEREST STATEMENT

The authors declare no conflicts of interest.

## ETHICS STATEMENT

Ethical approval for this study was obtained from Local Ethical Committee: COMITATO ETICO TERRITORIALE (CET) INTERAZIENDALE AOU CITTA' DELLA SALUTE E DELLA SCIENZA DI TORINO; reference # CET00057/2026.

## Supporting information

Supporting File 1.

## Data Availability

The data that support the findings of this study are available on request from the corresponding author. The data are not publicly available due to privacy or ethical restrictions.
