# Supplementary material for: A simple maximum intensity projection method for measuring proximal femoral anteversion: An observer reliability study
Source: J Exp Orthop. 2026 Oct 19;13(4):e70957. doi: 10.1002/jeo2.70957 (PMC13680545; doi:10.1002/jeo2.70957)
Supplement: Supplementary file 1 [file JEO2-13-e70957-s001.docx]

**Figure S1.** *The Strengthening the Reporting of Observational Studies in Epidemiology (STROBE) diagram for inclusion process*

**Figure S2.** *Bland–Altman plots of intraobserver agreement for Reader 1.*
Bland–Altman plots showing the agreement between repeated measurements for the five femoral neck anteversion measurement techniques: (A) Murphy, (B) Hernandez, (C) Waidelich, (D) Yoshioka, and (E) Maximum Intensity Projection (MIP). The central solid line represents the mean difference (bias), while the upper and lower dashed lines indicate the 95% limits of agreement (LoA).

**Figure S3.** *Bland–Altman plots of intraobserver agreement for Reader 2.*
Bland–Altman plots showing the agreement between repeated measurements for the five femoral neck anteversion measurement techniques: (A) Murphy, (B) Hernandez, (C) Waidelich, (D) Yoshioka, and (E) Maximum Intensity Projection (MIP). The central solid line represents the mean difference (bias), while the upper and lower dashed lines indicate the 95% limits of agreement (LoA).
